# Pharmaceutical Vehicles for Vaginal and Rectal Administration of Anti-HIV Microbicide Nanosystems

**DOI:** 10.3390/pharmaceutics11030145

**Published:** 2019-03-26

**Authors:** Letícia Mesquita, Joana Galante, Rute Nunes, Bruno Sarmento, José das Neves

**Affiliations:** 1 i3S—Instituto de Investigação e Inovação em Saúde, Universidade do Porto, 4200-135 Porto, Portugal; leticia.s.mesquita@gmail.com (L.M.); joana.galante@i3s.up.pt (J.G.); rute.nunes@ineb.up.pt (R.N.); bruno.sarmento@ineb.up.pt (B.S.); 2 INEB—Instituto de Engenharia Biomédica, Universidade do Porto, 4200-135 Porto, Portugal; 3 ICBAS—Instituto de Ciências Biomédicas Abel Salazar, Universidade do Porto, 4050-313 Porto, Portugal; 4 CESPU, Instituto de Investigação e Formação Avançada em Ciências e Tecnologias da Saúde, 4585-116 Gandra, Portugal

**Keywords:** antiretroviral drugs, dendrimers, dosage forms, HIV prevention, mucosal drug delivery, nanocarriers, nanomedicine, nanoparticles

## Abstract

Prevention strategies play a key role in the fight against HIV/AIDS. Vaginal and rectal microbicides hold great promise in tackling sexual transmission of HIV-1, but effective and safe products are yet to be approved and made available to those in need. While most efforts have been placed in finding and testing suitable active drug candidates to be used in microbicide development, the last decade also saw considerable advances in the design of adequate carrier systems and formulations that could lead to products presenting enhanced performance in protecting from infection. One strategy demonstrating great potential encompasses the use of nanosystems, either with intrinsic antiviral activity or acting as carriers for promising microbicide drug candidates. Polymeric nanoparticles, in particular, have been shown to be able to enhance mucosal distribution and retention of promising antiretroviral compounds. One important aspect in the development of nanotechnology-based microbicides relates to the design of pharmaceutical vehicles that allow not only convenient vaginal and/or rectal administration, but also preserve or even enhance the performance of nanosystems. In this manuscript, we revise relevant work concerning the selection of vaginal/rectal dosage forms and vehicle formulation development for the administration of microbicide nanosystems. We also pinpoint major gaps in the field and provide pertinent hints for future work.

## 1. Introduction

HIV/AIDS remains a huge healthcare burden. Despite continuous effort to circumvent the pandemic, the amount of new infections has been declining over recent years at a slower pace than required in order to meet the milestone set by the UNAIDS for 2020, i.e., less than 500,000 annual cases. During 2017 alone, 1.8 million people were estimated to be infected by HIV, mostly due to sexual exposure [1]. This alarming figure highlights that much more has to be done, particularly in the field of prevention. Alongside other approaches, topical pre-exposure prophylaxis (topical PrEP) holds considerable potential for tackling new HIV-1 infections [2]. This strategy comprises the use of microbicides, which can be defined as medical products intended to be administered in the vagina and/or rectum in order to avoid early steps of viral transmission upon sexual intercourse. The principle behind topical PrEP relies on the inhibition of HIV-1 at the mucosal level by one or several compounds, presenting more or less specific antiviral activity. Receptive partners are the ones potentially benefiting from microbicides, although reciprocal protection would also be desirable [3]. The field has come a long way but only mild success has been achieved. The most advanced microbicide in the development pipeline is the dapivirine (DPV) vaginal ring, which was shown to reduce HIV-1 acquisition in women by roughly 30% under placebo-controlled phase 3 clinical trials [4,5]. The product is now under regulatory evaluation and a final decision from the European Medicines Agency is expected soon [6]. Still, partial protection observed for more “conventional” products leaves a wide margin for advances in the development of microbicides.

Trailing on the exciting findings and achievements of nanomedicine, different nanotechnology-based microbicide systems were shown to present potentially advantageous features that could contribute to improved protection from HIV transmission. Several microbicide nanosystems, either with inherent antiviral activity or used as drug carriers, have been proposed over the years, often with promising pre-clinical results [7]. Still, one important feature has frequently been overlooked, namely the selection of dosage forms and the development of pharmaceutical vehicles (or platforms) that can incorporate microbicide nanosystems as active ingredients. The main objective is the development of final products that allow suitable vaginal and/or rectal self-administration by users. However, other questions can be further raised regarding functionality. For example, what are the interactions occurring upon incorporation (e.g., chemical or physical bonding between nanosystems and excipients from vehicles, structural changes to the matrix of polymeric vehicles, coating of nanosystems with components of vehicles) and how will these affect the behavior of nanosystems and vehicles, both during storage and in vivo? Do we want/need platforms that have additional roles beyond simple vaginal or rectal administration? Indeed, to which extent can a vehicle influence the original performance of nanosystems, especially in vivo? The intention of the present manuscript is to provide a brief and critical appraisal of the different vehicles that have been considered for vaginal and/or rectal administration of microbicide nanosystems, as well as hints for future work.

## 2. Microbicides for Preventing Sexual HIV Transmission

The idea behind the onset of modern microbicides is usually traced back to the early 1990s and, in particular, to the seminal commentary paper by Zena A. Stein [8]. In this, she called out to the need for the development of new methods that women could use in order to protect themselves against viral transmission. The following years saw the emergence of various drug candidates and vaginal microbicide products. This first generation of microbicides was characterized by the use of compounds featuring non-specific antiviral activity and simple formulations, mostly based on gels, although many other dosage forms have also been considered (e.g., creams, tablets, capsules, sponges, or films) [9]. The concept of using products already available in the market that could also present anti-HIV activity was particularly attractive. Nonoxynol-9, a non-ionic surfactant used in the composition of different vaginal spermicides and shown to present anti-HIV activity, was earlier suggested as a preferential target for development [10]. Results from clinical trials conducted during the late 1990s/early 2000s were, however, disappointing. Products based on surfactants and anionic/acidic polymers featuring non-specific antiviral activity not only lacked efficacy in protecting women against HIV acquisition but also, in some cases and paradoxically, increased transmission [11,12,13,14,15,16]. Additional studies confirmed that candidate microbicide compounds, such as nonoxynol-9 [17] and cellulose sulfate [18], can actually induce changes or even damage to the vaginal mucosa, thus facilitating HIV-1 transmission. Since then, a dramatic shift towards products based on potent antiretroviral drugs occurred in the field and particular emphasis was placed on safety evaluation. Additionally, formulation development of microbicide products benefited from increasing attention and was established as having a fundamental role in the performance of antiretroviral drug candidates [19].

Proof of a first partially successful microbicide was announced in 2010. Results from the CAPRISA 004 phase 2b clinical trial showed a 39% reduction in HIV-1 transmission in women using a 1% tenofovir (TFV) gel, as compared to placebo [20]. Partial protection was mainly attributed to poor adherence, which could have accounted for low or fluctuating levels of TFV throughout the timeframe of potential viral exposure and, thus, insufficient protection [21]. Recent evidence of differential drug metabolism by vaginal microbiota has been reported as another possibility [22]. Despite all the excitement around this first success, further studies testing similar TFV gels administered in alternative regimens failed to confirm the potential for protection [23,24]. Adherence issues were again held accountable [24,25].

Vaginal rings have the potential to abbreviate adherence issues observed with coitally-dependent products based on dosage forms, such as gels. This possibility motivated the development of the DPV ring, which allows maintaining sustained drug levels in the vagina for several weeks [26]. Somewhat surprisingly, data from two phase 3 clinical trials demonstrated only mild reduction (27–31%) in HIV acquisition by women [4,5]. Again, poor adherence was claimed as one of the main contributor to these results [27]. Intermittent use of the ring leads to a sharp decline in vaginal levels of DPV, which creates opportunity windows for viral transmission. Notably, adherence to product use is a key issue in microbicide protection. Post-hoc analysis of data from phase 3 clinical trials of the DPV ring indicates that higher protection rates were observed for women that used the product consistently [28]. Despite multiple factors being involved, acceptability and preferences regarding dosage forms and usage schemes are recognized as important in determining adherence [29]. Current thought in the field supports that a single product will not be enough to fulfil the requirements of a majority of women. Instead, a relatively vast panel of products from which to choose from, including multipurpose ones, will likely be required [30].

Microbicides for rectal use have followed on the footpath of vaginal products. Several antiretroviral drugs and dosage forms (e.g., suppositories or enemas) have been considered [31], although products based on TFV and presented as gels have been frequently selected as preferential. Some of the latter have reached as far as phase 2 clinical trials [32,33]. Product design has been regarded with equal or even greater importance than vaginal microbicides. The particularities of the colorectal compartment make it more susceptible to HIV transmission than the cervix/vagina [34] and a challenge to formulation. For example, the large surface area available for viral entrance that requires to be covered by a single product, the high susceptibility of the thin single columnar epithelium to toxic insult, or the possibility of extensive systemic absorption of the administered drug(s) are just some of the possible problems that need to be considered when developing a rectal microbicide [35].

## 3. Potential of Nanotechnology in Developing Microbicides

**Figure 1:**
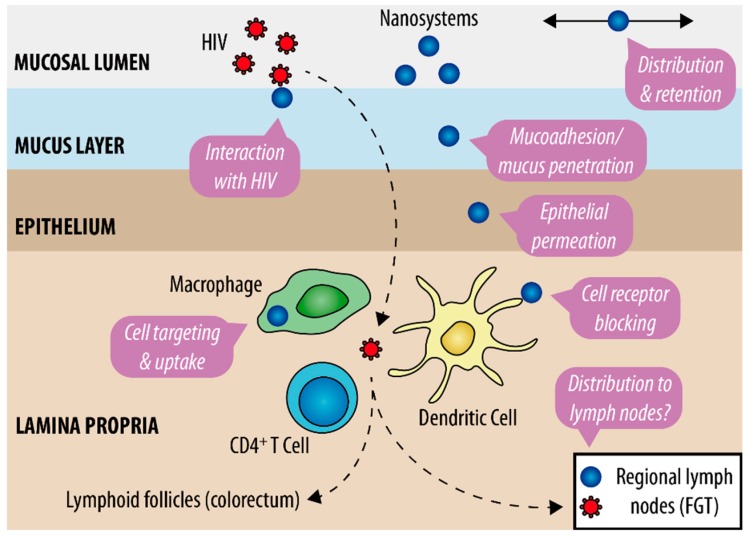
The potential of nanotechnology-based systems for microbicide development. Features of anti-HIV microbicide nanosystems at cervicovaginal or colorectal mucosal sites in the context of sexual HIV transmission are indicated in pink call-outs (see text for more details). FGT—female genital tract.

More than a trendy gimmick, the application of nanotechnology to the development of anti-HIV microbicides relies on solid research work, as previously revised by our group [7,36] and others [37,38]. General advantageous features are schematically presented in Figure 1. Although potentially applicable to both mucosal sites, work has been mostly conducted with the intention of vaginal use. Nanosystems intended for microbicide development may be classified in two major classes, as follows: (i) Nanosystems presenting intrinsic antiviral activity and/or competing with HIV for host targets and (ii) nanosystems acting as carriers for microbicide drugs [39]. In the first case, surface chemical functionalization of nanosystems is directly responsible for the inactivation of HIV or blockage of cell membrane receptors involved in viral infection. Dendrimers (often considered as polymers, not actual nanosystems) are the best representatives of this class, particularly SPL7013. This fourth-generation dendrimer is the active ingredient of VivaGel^®^ (Starpharma, Australia), mainly owing its anti-HIV activity to the direct interaction of terminal naphthalene disulfonate groups with gp120, a viral surface glycoprotein essential for cell infection [40]. Despite promising early results, the development of VivaGel^®^ as a microbicide has been discontinued following safety issues in clinical trials [41,42]. Meanwhile, other candidates have been under active development [43]. In particular, the G2-S16 dendrimer resulting from the systematic screening of a series of carbosilane dendrimers, proposed by Muñoz-Fernández and collaborators, was shown to be promising for further clinical testing [44,45]. Apart from dendrimers, other types of nanosystems with intrinsic activity have also been studied, namely oligomannose-coated gold nanoparticles (NPs) [46]. This type of nanosystems can block the C-type lectin receptor DC-SIGN present at macrophages and dendritic cells, which is involved in mediating HIV infection and translocation.

Interest in nanocarriers for the vaginal and/or rectal delivery of potent microbicide drugs has been growing over recent years. Fine tuning of the physicochemical properties of nanosystems, namely regarding size and surface properties, can provide active moieties with considerable advantage over “naked” counterparts (Figure 1). Apart from more general features, such as controlled drug release, protection of labile active molecules, or promotion of the solubility of hydrophobic compounds, nanocarriers may further (i) present enhanced distribution and retention along the mucosal site [47]; (ii) feature a variable ability to interact with mucus [48]; (iii) promote epithelial penetration and accumulation at mucosal tissues [49,50]; and/or (iv) target immune cells involved in viral transmission and increase intracellular drug accumulation [51]. One or more of these features should contribute to the improvement of local pharmacokinetics (PK) [52,53], i.e., prolong and/or increase drug levels at the organ/tissue/cell level and ultimately enhance protection from HIV transmission [54]. Antiretroviral drugs previously used in microbicide development, namely the viral transcriptase inhibitors TFV and DPV, have been selected as preferential compounds for assessing the potential of using nanocarriers [55,56]. Other more complex molecules have also been considered (e.g., fusion inhibitor peptides [57], RANTES analogues [58,59], or siRNA [60]). Polymeric NPs have been amongst the most popular nanocarriers used [61], although solid lipid NPs [62], liposomes [63], or nanolipogels [64] have been considered as alternatives. Furthermore, nanofibers attracted great interest as vaginal microbicides due to their ability to modulate drug release, provide fast distribution of incorporated compounds, and allow formulation of multiple active molecules in the same system [65].

Discussion on whether nanosystems should interact with mucus or not has generated substantial interest over recent years [66]. Contrary to the common idea that mucoadhesion is advantageous to improve mucosal residence, nanosystems presenting reduced size (typically under 500 nm or less) and an inert surface (usually conferred by dense coverage with 5–10 kDa polyethylene glycol (PEG)) can better traverse mucus and distribute throughout the vaginal and colorectal epithelial surface [67]. Furthermore, the ability of mucus-penetrating nanosystems to permeate mucus and reach areas closer to the epithelia, where mucus turnover is slower, can actually promote retention as compared to mucoadhesive counterparts [68]. Still, a case-by-case analysis as to which behavior may better sustain the residence and distribution of NPs following administration seems advisable, as shown by a recent report on the comparison between mucoadhesive and mucus-penetrating poly(lactic acid)-hyperbranched polyglycerol-based NPs [69]. Another issue needing further investigation is the ability of certain nanosystems to reach sites of interest beyond mucosae. For example, a few studies suggest that NPs can partially undergo cell-mediated or even free transport from the vagina to regional lymph nodes associated with the female mouse genital tract [70,71]. This may be relevant since HIV also endures a similar path following initial proliferation at the cervicovaginal mucosa [72]. Scanty evidence further indicates that the transport of vaginally administered NPs to the upper parts of the mouse genital tract may occur, albeit to a limited extent [73].

## 4. Vehicles for Microbicide Nanosystems

Pharmaceutical vehicles play an essential role in the delivery of active ingredients, encompassing both the general features of a defined dosage form and the individual properties of the specific formulation and manufacturing process. Broad definitions of “dosage form” adopted by regulatory bodies typically embrace the physical form of a product that contains one or more active ingredients and is intended to be administered to individuals in need [74,75]. For the purpose of this manuscript and from a technological perspective, microbicide nanosystems as a whole are considered as active ingredients. Various dosage forms have been traditionally considered for developing vehicles for vaginal and rectal drug administration and these have served as the usual starting point for the incorporation of nanosystems. Interested readers are further referred to compendia detailing the general principles of vaginal and rectal drug delivery and dosage forms [76,77]. Importantly, the design and production of vaginal or rectal vehicles should envision complying with the particularities of the mucosal site in which administration is intended. Many differences exist between both environments and some of the most relevant are summarized in Table 1. Safety is particularly relevant in the case of microbicides. Lessons learned the hard way in the early days of the field are now being translated into valuable guidance that aids in product development, particularly at preclinical stages [78]. Other features, such as manufacturing feasibility, cost, and acceptability by end-users must also be taken into consideration. Here we discuss the most relevant aspects of different vaginal and/or rectal vehicles, organized by dosage form, that were used for incorporation and delivery of microbicide nanosystems and detail on their relevance to the performance of such active ingredients.

### 4.1. Suspensions

Aqueous liquids are, by far, the most common vehicles in which microbicide nanosystems have been suspended and tested, particularly for in vivo studies. Fluidity of these systems can be seen as an advantage since it promotes wide and fast distribution along the mucosal site upon administration and is particularly important in the case of rectal microbicides [79]. However, the chief reason for their use probably relates to the fact that nanosystems are usually produced/synthetized in aqueous media and, indeed, originate nanosuspensions. Formal formulation efforts are usually minimal since convenience, rather than the achievement of a putative pharmaceutical product, usually drives selection of this type of vehicles. Thus, for the purpose of this work, all nanosuspensions are considered as de facto dosage forms. Saline-based buffers (approximately isoosmolal and with the pH around neutral) have been the most commonly used vehicles [52,53,80,81]. Indeed, particular attention should be given to pH and osmolality, as these factors are recognized to potentially affect safety [82]. While vehicles featuring pH around neutrality appear to be suitable for rectal vehicles, the choice of such low hydrogen-ion concentrations is related to the physiology of animal models typically used to test microbicides [83]. Deviations from the normal vaginal pH of women of reproductive age (4–5) could, however, potentially affect performance.

**Figure 2:**
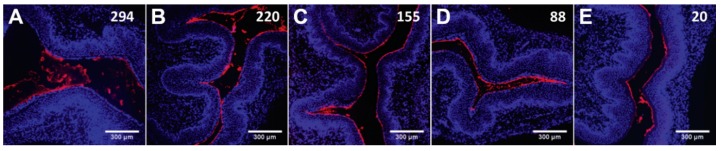
Vaginal distribution of fluorescent 100 nm mucus-penetrating particles administered in suspensions of varying osmolality. (**A**–**E**) Distribution of mucus-penetrating particles in transverse vaginal cryosections. Values of osmolality are presented in the individual images and have mOsm/kg as units. Images are representative of *n* ≥ 5 mice. Adapted from [84], Copyright (2013), with permission from Elsevier.

Vehicles matching physiological osmolality are usually regarded as suitable. Still, Ensign et al. [84] showed that hypoosmolal vehicles can actually enhance the distribution and retention of 100 nm mucus-penetrating NPs in the vagina, without apparent mucosal damage. These findings were explained by the promotion of faster transport of NPs across mucus and towards the epithelial surface, as driven by osmotic convection. The effect was pronounced even when a slight variation from isoosmolality was observed (Figure 2) [84]. The same group also studied the effect of rectally administered 60 nm mucus-penetrating NPs and found that osmolality affected distribution and retention, although the effect was more complex and dependent on ion composition [85]. For instance, potassium-based, but not sodium-based, had significant impact on osmotic convection when testing for osmolality at or below normal levels (20–300 mOsm/kg). This effect was attributed to the role of ion transport on water absorption at the colon. While sodium promotes water uptake by tissue, the opposite is true for potassium [85]. Overall, these studies suggest that the performance of microbicide nanosystems can be enhanced or impaired by proper selection of liquid vehicles and at least their qualitative and quantitative composition should be reported.

A final aspect of liquid vehicles that should not be underestimated concerns their general low vaginal and rectal retention following application. Leakage is typical and, in the case of rectal administration, extensive if voluntary anal sphincter closure by users is not observed. The presence of material in the rectal ampulla induces an urge to defecate that needs to be firmly opposed upon administration. This problem is particularly noteworthy with increasing volumes being administered. Fractionated administration in a supine position may help mitigate leakage.

### 4.2. Gels

Gels are among the most popular dosage forms for vaginal and rectal drug delivery, largely due to their technological versatility, acceptability by users, and low production cost. These have been widely explored in microbicide formulation [86]. The semi-solid nature of gels as well as the mucoadhesive properties of various gelling agents may help with improving the vaginal and colorectal residence of nanosystems. Not surprisingly, this was the chosen dosage form selected for the SPL7013 dendrimer in order to obtain VivaGel^®^. The gel is based on carbomer 941 and is reported to contain 1% (*w*/*w*) of glycerin and propylene glycol, although fully detailed composition and basic properties (pH, osmolality, viscosity, dendrimer release, etc.) are not available [87]. A role for the gel on safety issues detected during clinical testing was not evident [41,42].

Selection of gel bases for the incorporation of microbicide nanosystems has been typically made in an empirical fashion. Most commonly used gelling agents include different carbomers [88] and hydroxyethylcellulose [44]. The common use of this last cellulose derivative in microbicide development has its origins in the established safety of the hydroxyethylcellulose-based formulation “Universal Placebo” [89]. However, a more systematic approach to the development of gels as platforms for microbicide nanosystems is recommended. Interactions between both are particularly relevant and may potentially impact the final protection outcomes. For example, Wang et al. [90] developed efforts in order to formulate different gels containing liposomes loaded with the microbicide candidate octylglycerol. Among other effects, the addition of gelling agents, such as carbomers, led to a mild reduction of the in vitro efficacy of octylglycerol-loaded liposomes against HIV-1. This effect was attributed to a reduction in the amount of octylglycerol that could be released, due to the increased viscosity. Still, the setup used for testing efficacy may have overlooked another relevant aspect of gels, namely the ability to provide a barrier to effective HIV diffusion. In a study by Lara et al. [91], the incorporation of microbicide polyvinylpyrrolidone (PVP)-coated silver nanoparticles (30–50 nm) into a carbomer-based commercial gel (Replens™, Lil’ Drug Store Products, USA) slightly improved the inhibition of HIV-1 infection when the product provided a layer on top of cervical explants. The viscous layer presumably provided an additional barrier for virions to cross until reaching target cells present in tissue. Although limited animal data reported previously by other researchers appear to support this possibility [92], it is debatable for how long could such a barrier actually impact viral transmission. Additional research on this topic is required.

### 4.3. Thermosensitive Systems

Thermosensitive systems envisioned for vaginal or rectal drug administration are typically presented as liquid dosage forms that present the ability to undergo sol-gel transition at just below body temperature, originating semi-solid gels [93,94]. Rationale for use relies on their ease of administration and a wide initial distribution of a fluid along the mucosal cavity, while a semi-solid (often incorporating mucoadhesive polymers) provides improved local retention. Thermosensitive systems considered for vaginal and rectal drug delivery have been almost exclusively based on poloxamers due to their well-known temperature-dependent sol-gel transition, regulatory status (currently used in different pharmaceutical products), and excellent safety profile [95]. The utility of thermosensitive dosage forms is established for mucosal drug delivery, but their ability to influence the performance of nanosystems is not well understood.

Thermosensitive systems have been preferred for the incorporation and administration of microbicide drug-loaded NPs [96]. From a simple technological perspective, incorporation of nanosystems into a fluid system at room temperature is easier than in an already pre-formed gel. However, the possibilities and pitfalls for microbicide NPs-in-thermosensitive systems may extend beyond that. For example, drug release and gelation temperature may be affected. Timur et al. [97] found that the incorporation of TFV-loaded chitosan NPs (≈545 nm) into a poloxamer-based thermosensitive vehicle allowed a mild delay in the release of the drug, as compared to NPs in suspension or the same thermosensitive platform containing free TFV. The gelation temperature was also affected by the incorporation of NPs [97].

One concern of using thermosensitive systems relates to the possibility that NPs are not able to escape the structured matrix formed upon sol-gel transition, thus affecting performance. Although impossible to generalize, Date et al. [98,99] showed that both poly(lactic-*co*-glycolic acid) (PLGA)- and cellulose acetate phthalate (CAP)-based NPs (around 80–100 nm) were able to be released from a poloxamer-based thermosensitive vaginal gel at 37 °C and be taken up by epithelial cells, as shown using an in vitro cell-based model. In the particular case of efavirenz (EFV)-loaded CAP NPs, the antiretroviral activity was demonstrated to be maintained following incorporation into the thermosensitive vehicle [99]. Importantly, the same thermosensitive gel, containing rilpivirine-loaded PLGA NPs (66 nm), was fully or partially (50%) effective in preventing HIV-1 transmission in a humanized mouse model when administered at 90 min or 24 h, respectively, before intravaginal viral challenge [54]. Although drug-loaded NPs in suspension were not actually tested in vivo, authors claimed that the protection observed even at 24 h post-administration was due to the extended residence of nanosystems provided by the thermosensitive vehicle. Microscopy analysis of vaginal sections, collected from animals treated intravaginally with fluorescent NPs-in-thermosensitive systems, indeed provided evidence that, although residual, NPs could still be found at 24 h post-administration. A subsequent study using the same thermosensitive vehicle to deliver tenofovir disoproxil fumarate (TDF)-loaded PLGA NPs (≈150 nm) to humanized mice also provided evidence of complete protection up to at least 24 h post-administration [100]. Again, the lack of a control group using drug-loaded NPs in a liquid vehicle limited the assessment of the true role of the thermosensitive system in the protection outcomes.

The use of NPs-in-thermosensitive vehicles seems to be particularly interesting for rectal use. Still, no specific study is available for the purpose of microbicide development. Apart from increased residence, wide distribution of microbicide nanosystems in the colorectum may be challenging. As stated previously, the liquid state preceding gel formation may allow enhanced distribution immediately upon intrarectal administration. Preliminary data from our group support that PLGA NPs (≈180 nm) administered to mice were able to provide roughly the same extent coverage of the colon when incorporated into phosphate buffered saline or a poloxamer-based enema [101]. Residence was enhanced in this last case, presumably due to the formation of a gel at body temperature.

### 4.4. Vaginal Films

Pharmaceutical films comprise thin, soft, flexible, and often translucent solid strips of polymeric nature, in which active ingredients are dissolved or dispersed [102]. Solvent-casting is the preferred technique for manufacturing films, although hot-melt extrusion can also be used. Upon contact with vaginal fluids, films typically dissolve rapidly and originate a low volume and gel-like fluid. However, films can also be designed to disintegrate slowly. Their use for vaginal drug administration, particularly in microbicide development, is well documented and supported by features such as the ability to (co-)formulate multiple drugs, the established technology, and good stability upon storage, among others [103,104]. Acceptability by women is usually high since the films are portable, easy to administer without the need of an applicator (contrary to liquids and semi-solids), and known to originate little vaginal leakage [105]. Although not so widely used as other vaginal dosage forms, a few contraceptive vaginal films are currently commercially available in selected countries.

The concept of using films as platforms for buccal/oral administration of nanosystems, namely drug-loaded polymeric NPs, has been around for over a decade [106], but its application to the development of vaginal microbicides is recent. General advantages for using films, namely the ones indicated above, continue to be valid, while more specific others may further apply [107]. In particular, the solid nature of films largely restrains drug leakage from nanosystems throughout storage, as well as other potential changes and microbial contamination due to the low amounts of water present (usually less than 10%). The tight polymeric matrix surrounding nanosystems also limits phenomena such as particle aggregation and segregation that can originate colloidal instability. Such possibilities cannot be ruled out for liquid/semi-solid systems and, indeed, often lead to stability issues [108]. The incorporation of NPs is usually conducted by mixing with an aqueous dispersion of film ingredients before casting and drying of films, but other strategies may also be adopted. For example, our group recently proposed a “sandwich-like” configuration film comprising TDF/emtricitabine-loaded Eudragit^®^ L 100 NPs (≈400 nm) entrapped between two individual sheets of poly(vinyl alcohol)/hydroxypropyl methylcellulose (PVA/HPMC) film, previously prepared by solvent-casting [109]. Assembly of the final film was achieved by compression and the obtained system allowed for decreasing in vitro burst-release of both drugs.

**Figure 3:**
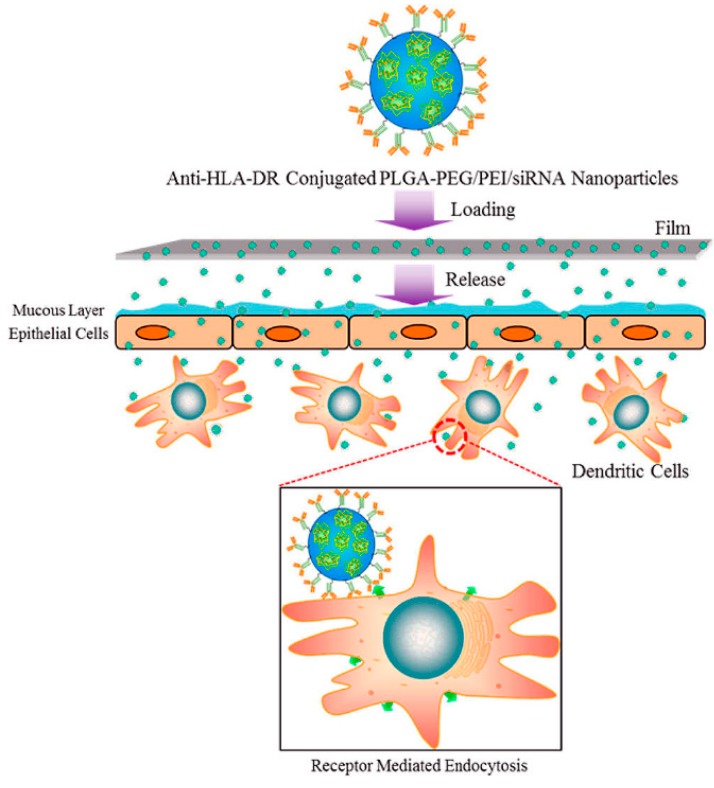
Schematic representation of targeted siRNA-loaded NPs formulated into a biodegradable film for targeting siRNA delivery into HLA-DR+ dendritic cells, using a co-culture cell model. Targeted siRNA-loaded NPs are homogeneously dispersed in a biodegradable film and, upon administration, the film is expected to disintegrate within the vaginal lumen, allowing siRNA-loaded NPs to penetrate across the vaginal mucosa and deliver siRNA in a targeted manner to HLA-DR+ mKG-1 dendritic cells. Reprinted with permission from [110]. Copyright (2015) American Chemical Society.

Gu et al. [110] were the first to describe a NPs-in-film system for the development of a microbicide product. They produced siRNA-loaded, anti-human leukocyte antigen-DR (HLA-DR) antibody-functionalized PEG-PLGA-based NPs, with around a 230 nm mean diameter, and associated it to PVA/λ-carrageenan-based films using solvent-casting. The siRNA used was intended to silence the translation of SNAP-23, a host protein associated with HIV exocytosis, while the incorporation of the antibody envisioned targeting HLA-DR+ dendritic cells. Although successful, silencing was reduced when NPs-in-films were compared to NPs in aqueous suspension, thus suggesting that the film matrix could reduce the transport of NPs across the epithelial cell monolayer and towards target dendritic cells in an in vitro co-culture cell-based model (Figure 3). Even if these data seem little encouraging, the actual value of microbicide NPs-in-films is more likely to be observed in vivo.

**Figure 4:**
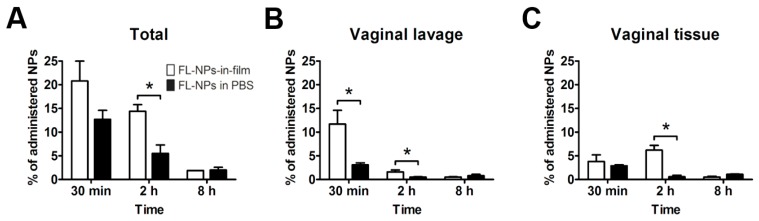
Distribution of fluorescent NPs (FL-NPs) at different times after intravaginal delivery in phosphate buffered saline (FL-NPs in PBS) or film (FL-NPs-in-film). Results are expressed as the (**A**) total amount of recovered particles or fractions retrieved from (**B**) vaginal lavage and (**C**) vaginal tissues. Columns and bars stand for mean and standard error of the mean values, respectively, and (*) indicates *p* < 0.05 (Student’s *t*-test; *n* = 3). Adapted from [111], Copyright (2016), with permission from Elsevier.

Our group has been particularly engaged in testing microbicide NPs-in-films in animal models. For example, we developed a hybrid film, based on PVA/HPMC, containing TFV (unassociated to NPs) and EFV-loaded PLGA NPs (≈275 nm) dissolved/dispersed in the film matrix [111]. Biodistribution experiments showed that, although substantial initial leakage occurred in all cases, the overall vaginal residence of NPs was significantly higher upon incorporation into films (Figure 4). The system containing TFV and EFV-loaded PLGA NPs allowed higher reaching and prolonged levels for both transcriptase inhibitor drugs in vaginal tissues and fluids following administration to mice, as compared to their administration in a liquid vehicle. Additionally, the tandem association of EFV to NPs and film allowed an additional improvement of local PK, as compared to the drug incorporated directly into the film matrix. These data appear to support that, indeed, the combination of NPs and films may provide an advantageous system for sustaining local levels of microbicide drugs. Furthermore, NPs-in-film tested were shown safe, following daily intravaginal administration to mice for 14 days [111,112]. Contrasting with the previous data, Srinivasan et al. [113] did not find the use of IQP-0528-loaded NPs-in-film to be advantageous, as compared to the free compound directly incorporated into the film. In fact, vaginal drug levels were shown to be slightly higher for this last formulation when administered intravaginally to pigtailed macaques. One possible reason may have been related with the intentionally slow and only partial release of IQP-0528 from NPs-in-film, as shown in vitro, which could have limited the ability of the drug to reach vaginal fluid and tissues before bulk vaginal leakage of the films [113]. Overall, these results seem to reinforce the need for fine tuning the technological properties of microbicide NPs-in-film systems.

### 4.5. Fiber Mats

**Figure 5:**
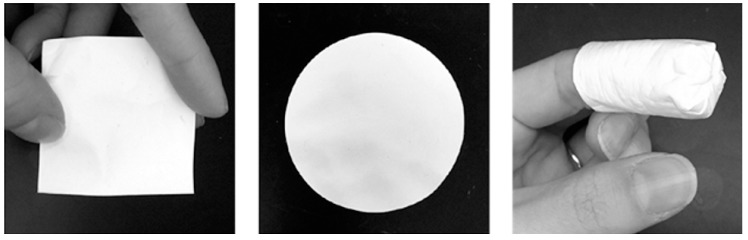
Examples of differently shaped, human sized PVA nanofiber mats (fiber cross-section diameter of ≈200–300 nm and mat thickness of 50–220 μm according to Krogstad et al. [119]). *Left*—square (25 cm^2^); *center*—circle (diameter = 5 cm); *right*—capped tube (length = 4 cm, diameter = 2 cm). Adapted from [118] under the terms of the Creative Commons Attribution License 4.0 (Copyright 2018 Laborde et al.; doi:10.1371/journal.pone.0204821).

Drug-loaded fiber mats or membranes captured substantial interest over recent years for developing vaginal microbicides [114]. Their rectal use has not yet been considered. Depending on the cross-section dimensions, fibers can be classified as nanofibers and constitute actual microbicide nanosystems. One of the advantages of fibers is the possibility of being used as such, namely in the form of differently shaped and sized mats (Figure 5). Similar to vaginal films in general appearance, but typically opaque, fiber mats are produced by electrospinning and present sufficient versatility to incorporate multiple drugs (namely for other applications, such as contraception) in various configurations. The fine-tuning of antiretroviral drug release from fibers and/or composite mats is also relatively simple to achieve [115]. Drug release is associated with fiber composition and structure and can be triggered by environmental changes pertinent to sexual intercourse, namely happening upon ejaculation (e.g., rising pH or the presence of components from semen) [116,117]. Depending on specific characteristics, namely concerning the ability to dissolve and the perception of which shape is easier to insert in the vagina, fiber mats appear to be well accepted by potential female users of microbicides [118].

Fibers themselves can be used as vehicles for the administration of microbicide nanosystems. The concept is quite recent but available studies suggest great potential. Krogstad et al. [120] first proposed the use of two types of nanofibers, based either on PVA (≈250 nm cross-section diameter) or PVP (≈300 nm cross-section diameter), two mucoadhesive polymers, for the incorporation of PLGA-based NPs (≈170–180 nm). Composite nanofiber mats were rapidly wetted and disintegrated within a few seconds when exposed to an aqueous environment. Complete dissolution and release of over 85% of the content in NPs was observed to occur within 30 min under in vitro conditions. However, significant changes in size, polydispersity and zeta potential of the NPs were observed, particularly when PVA was considered, which suggests that coating with material from nanofibers occurs upon particle release. In an expanded series of distribution and retention studies using mice, composite nanofibers were not only able to provide extensive coverage of the vaginal mucosa with NPs, but also to significantly reduce leakage [120]. Mean recovery of NPs was around 45–50% from the total administered dose at 24 h, in the case of composite nanofibers, contrasting with under 5% for NPs administered in suspension. Strikingly, 40% of NPs were still recovered after three days in the case of PVA composite nanofibers. These results translated into enhanced PK when etravirine-loaded PLGA NPs incorporated into PVA nanofiber mats were tested. For example, fibers provided around 13-times higher drug levels in lavage up to three days post-administration, but roughly the same concentrations in vaginal tissue in the same time-frame, as compared to drug-loaded NPs in suspension and estimated by the area-under-the-curve values [120]. Relatively poor accumulation at tissues suggests that relatively rapid release of etravirine from NPs, low mobility of NPs towards the mucosa, or both, occurred. Still, overall data seems to support the utility of PVA nanofibers in enhancing the residence and PK of microbicide NPs.

An interesting approach to the use of fibers for the administration of microbicide NPs has been recently proposed by Kim et al. [121]. The researchers proposed electrospun fibers composed of a non-dissolving, pH-responsive polyurethane co-polymer, namely PEG-1,4-bis(2-hydroxyethyl)piperazine-4,4′-methylenebis(phenyl isocyanate)-propylene glycol, to be used for the controlled release of NPs. Fibers were not produced with the intention of constituting the actual dosage form, but rather to serve as a release limiting membrane that could be useful, as suggested by the authors, in the design of vaginal rings. The concept behind the responsive membrane was again based on the shift from acidic (4–5) to slightly alkaline (>7) values of pH observed in the vagina upon ejaculation. Membranes presenting in the dry state with a mean pore size and fiber diameter of 2.3 μm and 0.9 μm, respectively, were able to swell differently in a simulated vaginal fluid, depending on pH. This effect was shown to be mostly related to the differential protonation of the 1,4-bis(2-hydroxyethyl)piperazine (HEP) group, which could additionally lead to variable electrostatic interactions with charged NPs. In practice, and although pH-dependent behavior was also observed for a control polyurethane co-polymer without HEP, membranes allowed roughly 0% and 60% in vitro permeation after 24 h of model polystyrene NPs (≈200 nm) when tested at pH 4.5 and 7.0, respectively. Control of permeation for solid lipid NPs containing anti-CCR5 siRNA and presenting a mean diameter of around 270 nm was milder (around 30% and 60% at pH 4.5 and 7.0, respectively) [121]. Overall, developed fiber mats could be useful in the design of “smart” dosage forms able to release the majority of microbicide NPs only upon potential contact with the virus.

### 4.6. Other Potential Systems

Various additional dosage forms present the potential to be used for the vaginal or rectal administration of microbicide nanosystems. Strangely enough, some more “classical” vaginal and rectal dosage forms, such as tablets, soft capsules, inserts (suppositories and ovules), creams, and ointments, have not been described in the literature as vehicles for microbicide nanosystems. Technological feasibility to incorporate nanosystems in such platforms is recognized, but specific demonstration studies still need to be performed for nanotechnology-based microbicides. Foams may also be feasible and interesting platforms for the incorporation of microbicide nanosystems. In the only relevant study available, Vedha Hari et al. [122] reported on the basic technological features of EFV-loaded Eudragit^®^ E 100 NPs (≈110–260 nm) incorporated into a foam formulation. The system was intended for vaginal use but the presence of sodium lauryl sulfate as a foaming agent may compromise the safety of the putative microbicide product.

Nanotechnology-based microbicides have been developed essentially as on-demand products, i.e., requiring administration within minutes to hours of coitus. Although challenging from a technological viewpoint, the development of coitally-independent delivery platforms that allow for prolonged intravaginal residence and sustained release of microbicide nanosystems could be an interesting new approach. For example, vaginal ring technology has come a long way over the last decade and appears to offer enough versatility for the appropriate incorporation and release of nanosystems [123]. Although the pH-sensitive polyurethane fiber membrane described in the previous sub-section was suggested for the design of rings, only the concept was presented and sustained release over at least several days was not demonstrated [121]. Alternatively, simpler rings based on designs previously explored for macromolecule delivery using, for example, pod-inserts [124] or exposed cores [125] could be seen as promising, but pioneering work is required.

## 5. Conclusions and Future Perspectives

The currently available body of knowledge supporting the development of nanotechnology-based microbicides is extensive and relies on solid grounds. Nanosystems with intrinsic activity against HIV-1 led the field early on (even reaching clinical testing), but have recently been overcome by the promising results obtained for antiretroviral drug-loaded nanocarriers. Still, much more has to be done until a robust candidate nanosystem can be targeted for advanced pre-clinical and clinical testing. Among different issues, suitable vehicles that can enable convenient administration of microbicide nanosystems require development. Several dosage forms have been proposed and tested. Proper design and characterization is paramount in order to guarantee that the original features of nanosystems are at least maintained or, ideally, enhanced. Understanding the interactions between microbicide nanosystems, administration platforms, and the mucosal environment is essential and not always broadly assessed in the work conducted so far. Thermosensitive systems, vaginal films, and fiber mats have been recently studied and hold the potential to provide improved distribution and retention of nanosystems following administration and, ultimately, improve PK at the mucosal level.

Still, many questions remain and various possibilities need to be explored. The stability of nanotechnology-based microbicides is usually overlooked, namely when incorporated into pharmaceutical vehicles. A particular concern has to deal with the release kinetics of drug payloads from nanocarriers and into the matrix of vehicles during manufacturing and storage. Product complexity and a lack of standards for evaluation pose important challenges that need to be addressed. These often translate into costly manufacturing and control, which are incompatible with achieving affordable microbicides. Another important issue in microbicide product development concerns users’ preferences and acceptability. Products that meet women’s and men’s expectations are more likely to be consistently used and, thus, be able to provide proper protection. Furthermore, the idea of investing all efforts in developing a single microbicide product for universal use is illusive and outdated. The possibility to choose from a set of products which better suit individual lifestyles and situations seems to be paramount. The development of behaviorally congruent microbicides, i.e., products that can be incorporated in widespread practices associated with sex (e.g., the use of cleansing enemas in preparation for anal sex), is another trend in the microbicides field [126]. Overall, such principles need to be introduced early in the design process of microbicides, including those based on nanotechnology. The development of dual compartment (or dual chamber) microbicide formulations, i.e., for both vaginal and rectal use, has been pursued in the past as a way to meet user’s needs and improve adherence [127]. Such strategy would also be interesting for developing nanotechnology-based microbicides, since nanosystems may hold the potential for both vaginal and rectal protection from viral transmission [49]. However, physiological differences between the two anatomical sites often make it difficult to design products that fit dual usage and, thus, the concept has been somewhat left behind over more recent years. A final word for the regulatory concerns of developing nanotechnology-based microbicides is as follows: While still waiting for the first microbicide product to be approved, general guidance from medical agencies on nanomedicines [128,129,130] and microbicides [131], as well as the particular cases of VivaGel^®^ and the DPV ring, should be considered as relevant standards.

## Figures and Tables

**Table 1:** Typical characteristics of vaginal and rectal mucosae (revised in [35,76]).

Characteristics	Vaginal Mucosa ^a^	Rectal Mucosa
Extension ^b^	9–12 cm	15–20 cm ^c^
Surface area	65–165 cm^2^	200–400 cm^2^
Epithelium	Stratified squamous	Simple columnar
pH of mucus	4–5	7–8
pH buffering capacity of mucus	Low	Low
Typical volume of mucus	0.5–1 mL ^d^	1–3 mL
Mucin concentration in mucus	1–2%	<5%
Osmolality of mucus	Nearly isoosmolal ^e^	Nearly isoosmolal ^e^
Enzymatic activity	Low	Medium
Microbiota composition	Lactobacilli dominant	Variable
Involuntary motility	Low	Medium to high

^a^ Considering healthy women of reproductive age; ^b^ at the longest axis; ^c^ total extension of the colorectum is around 150 cm; ^d^ largely increased upon sexual stimulation; ^e^ as compared to the osmolality of blood plasma (≈290 mOsm/Kg).
